# The Effect of Ascorbic Acid and Garlic Administration on Lead-Induced Neural Damage in Rat Offspring’s Hippocampus

**Published:** 2013-02

**Authors:** Akram Sadeghi, Alireza Ebrahimzadeh Bideskan, Fatemeh Alipour, Alireza Fazel, Hossein Haghir

**Affiliations:** 1 Department of Anatomy and Cell Biology, School of Medicine, Mashhad University of Medical Sciences, Mashhad, Iran; 2 Medical Genetic Research Center (MGRC), School of Medicine, Mashhad University of Medical Sciences, Mashhad, Iran

**Keywords:** Ascorbic acid, Garlic, Hippocampus, Lead

## Abstract

***Objective(s):*** The aim of this study was to investigate ascorbic acid and garlic protective effects on lead-induced neurotoxicity during rat hippocampus development.

***Materials and Methods:*** 90 pregnant wistar rats were divided randomly into nine groups: 1- Animals received leaded water (L). 2- Rats received leaded water and ascorbic acid (L+AA). 3- Animals received leaded water and garlic juice (L+G). 4-Animals received leaded water, ascorbic acid and garlic juice (L+G+AA). 5- Rats treated with ascorbic acid (AA). 6- Rats treated with garlic juice (G). 7- Rats treated with ascorbic acid and garlic juice (AA+G). 8- Rats treated with tap water plus 0.4 ml/l normal hydrogen chloride (HCl) and 0.5 mg/l Glucose (Sham). 9- Normal group (N). Leaded water (1500 ppm), garlic juice (1 ml/100g/day, gavage) and ascorbic acid (500 mg/kg/day, IP) were used. Finally, blood lead levels (BLL) were measured in both rats and their offspring. The rat offspring brain sections were stained using Toluidine Blue and photographed. Dark neurons (DNs) were counted to compare all groups.

***Results:*** BLL significantly increased in L group compared to control and sham groups and decreased in L+G and L+AA groups in comparison to the L group (P<0.05). the number of DNs in the CA1, CA3, and DG of rat offspring hippocampus significantly increased in L group in comparison to control and sham groups (P<0.05) and decreased in L+G and L+AA groups compared to L group (P<0.05).

***Conclusion:*** Garlic juice and ascorbic acid administration during pregnancy and lactation may protect lead-induced neural damage in rat offspring hippocampus.

## Introduction

Although lead is a useful metal in life and is used in modern industries and agriculture, it is one of the most toxic heavy metals for body and its poisoning is known as an important public health problem (1-6). Several researches have demonstrated that lead can cause neurological, hematological, gastrointestinal, reproductive, circulatory, and immunological disorders (7, 8). 

Lead can enter body mainly via eating, drinking or inhalation and transport to many tissues such as kidney, liver, bones and brain. As estimated by the World Health Organization (WHO), the total lead intake from food (nonoccupationally exposed) by an adult is in the range of 26–282 µg/day in various countries (9). Lead passes through the blood-brain barrier (BBB) rapidly and concentrates in the brain, and can readily cross through the placenta in lead- exposed pregnant mothers (8, 10). In this regard, pregnant women are the group at increased risk of lead exposure to their fetus. There are obvious evidences indicating that lead exposure during pregnancy and lactation has irrecoverable effects on later cognitive and behavioral development (7, 8). Because, in these developmental stages, the brain is in a state of rapid growth, and toxic metals exposure such as lead may result impairment of later cognitive functions (11, 12). Despite the strong evidences indicating the association between lead exposure and behavioral and cognitive impairments, the mechanisms by which lead causes these disorders remain poorly understood.

 Dark neuron is a unique type of cell degeneration in which characterized by cytoplasmic and nuclear condensation, neuron shrinkage, and failure of cell functions (13, 14). Dark neurons (DNs), in histological studies, are recognized by hyperbasophilia, hyperargyrophilia, and hyper-electron density properties. These morphological neurotoxicity changes are observed in stroke, head trauma, hypoglycemia, extreme seizures, and heavy metal neurotoxicity (15). At least four types of “dark” neurons are accepted: the Huntington type (observed in a mouse model of experimental Huntington disease), the artefactual type (produced by unintentional postmortem mechanical injuries of various kinds), the reversible type (early stages of hypoglycemic, epileptic or ischemic injury) and the irreversible type (late stages of hypoglycemic, epileptic or ischemic injury) (16).

There are several investigations indicating the lead-induced changes in hippocampal NMDA (N-Methyl –D – Aspartate) receptor subunits mRNA (16). It has been reported that glutamate release and neuronal transmembrane ion fluxes could be the perturbation leading to dark neuron formation. Regarding mentioned researches in this work, we investigated the numerical density of DNs in hippocampus proper and dentate gyrus of developmentally lead exposed rat offspring as a predictor of neuronal injury (15). 

There are some suggested mechanisms for the production of Dark neuron. On the other hand, *in vivo* and in vitro studies suggest that, lead exposure may increase the production of reactive oxygen species (ROS) and alteration of antioxidant defense systems in animals (17-19). Therefore, antioxidants such as Vitamin C (Ascorbic Acid) act as a free radicals and ROS scavenger, and reduce the possibility of lead interacting with critical bimolecular and factors inducing oxidative damage (20-22). In addition, many researchers reported that vitamin C is a chelating agent in the treatment of lead toxicity (23-26).

Garlic (*Allium sativum*) is used throughout history for both culinary and medicinal purposes such as antimicrobial, antithrombotic, antihypertensive, antihyperglycemic and antihyperlipemic (27, 28). This medicinal plant has several active components with well-known biological functions (29, 30).

It is said that aqueous garlic extract acts as an antioxidant by scavenging ROS, enhancing cellular antioxidant enzymes superoxide dismutase, Catalase, Glutathione peroxidase and inhibits lipid peroxidation and activation of oxidant induced transcription factors (21, 27, 31). 

In this regard, the aim of the present study was to investigate the possible protective effects of garlic juice and acid ascorbic on lead-induced dark neuron. 

## Materials and Methods

*Animals*

Ninety virgin adult female Wistar rats (six week old, and weighing 250-300 g) were used in the present study. Through the experiment, the animals were maintained at the animal house under controlled conditions (12 hr light and dark cycle, 22°C and 60% relative humidity) with laboratory chow and water provided ad libitum (32). 

all procedures involving animals were performed in accordance with the Guideline for Care and Use of Laboratory Animals of Mashhad University of Medical Sciences, Mashhad, Iran.

*Study design and experimental groups*

Female rats were mated with males of the same strain. The day on which spermatozoa were found in the vaginal smear was designated as gestational day 0 (GD0). Then, the pregnant rats were divided into 9 groups randomly (n=10 in each group) as follows: 

1- lead-exposed(L)group; the animals were treated with 1500 ppm lead acetate in drinking water starting at GD0. The lead exposure regimen was chosen based on a previous study (33). 

2- lead + ascorbic acid (L+AA) group; the animals were treated with 1500 ppm leaded-water and ascorbic acid (500 mg/kg) via intraperitoneal injection (IP) once a day (22).

3- lead + garlic juice (L+G) group; the animals were received leaded-water and fresh garlic juice (1ml /100g/body weight) by gavage once a day (21).

4- lead + ascorbic acid +garlic (L+AA+G) group; the animals were treated with leaded water and ascorbic acid (500 mg/kg) via intraperitoneal injection and fresh garlic juice (1ml /100g/body weight) by gavage once a day (21).

5- Ascorbic acid (AA) group; the animals were treated with ascorbic acid (500 mg/kg) via intraperitoneal injection once a day.

6- Garlic (G) group; the animal were treated with fresh garlic juice (1ml /100g/Body weight) once a day by gavage.

7- Ascorbic acid + garlic (AA+G) group; the animal were treated with 500 mg/kg via intraperitoneal injection and fresh garlic juice (1ml /100 g/body weight) by gavage once a day.

8- Sham (Sh) group; animals were treated with tap water plus 0.4ml/l normal hydrogen chloride (HCl) and 0.5 mg/l Glucose.

9- Normal (N) group; animals were administrated with tap-drinking water.

All the treatments were continued during pregnancy and lactation (postnatal day 21=P21). After P21, pups were kept in the treatment regimens until P50. 

*Preparation of leaded water*

For the preparation of 1500 ppm leaded water, 30 g lead acetate, 8cc normal HCl (to avoid lead precipitation) and 10g glucose (for favorite taste) were dissolved in 20 liters of tap water.

*Source of garlic*

Fresh garlic bulbs were collected from a natural habitat around Mashhad during June to August 2011, and identified by botanists in Ferdowsi University of Mashhad, Iran and a voucher number deposited (FUMH: 39493). 

*Preparation of garlic juice*

To prepare garlic juice, garlic bulbs were separated, peeled and washed with distilled water. After drying in a shed, the clean garlic bulbs were crushed with an electric grinder and the extract was decanted carefully through muslin cloth (21). 

*Blood lead level measurement*

At the end of the experiment, the young pups were deeply anesthetized with chloroform and blood sample was taken transcardinally. To measure lead level in whole blood samples, a Perkin-Elmer Model 3030 atomic absorption spectrophotometer with a Perkin-Elmer HGA (Heat Graphite Atomizer) 400 graphite furnace and hydride system MHS 10 was used together with HCL (Hallow Cathode Lamp) and EDL (Electrode Discharge Lamp) for metal measurement of even low levels. Blood was diluted 1:10 with Triton X-100, with the addition of a matrix modifier containing ammonium phosphate monobasic and magnesium nitrate. All specimens were run in batches which included standard methods for calibration (Table 1). BLL was measured in each animal group before and after interventions in mothers (rats) as well as their offspring at the end of the experiment (P50). 

*Histological method*

At the end of treatment, the young pups were deeply anesthetized and their brains were removed carefully, washed in normal saline and fixed in normalized fixative containing 10% formaldehyde in 0.01 M phosphate buffered saline (PBS) overnight at room temperature. After fixation, the specimens were dehydrated with an ascending ethanol series, cleared with xylen and embedded in paraffin. The brain tissue blocks were cut into 5μm transverse serial sections and stained with Toluidine blue (32).

**Table 1:** Atomic absorption spectrophotometer system calibration

Element	Pb	Current	8 mA
Wavelength (nm)	283.3	Slit width	1.00 nm
Recovery (%)	109	Drying	150ºC
Detection Limit (ppb)	0.8	Atomization	1,400ºC
Accuracy (%)	99.4	Precision (%)	3.44
Argon Gas	99.99%		

*Quantiﬁcation of DNs*

The DNs were identified microscopically by cytoplasmic and nuclear condensation, shrinkage and hyperbasophilia properties in the hippocampal pyramidal cells and dentate gyrus granular cells. 

The sections were scanned and photographed using a light microscope with a ×40 objective lens (UPlan FI, Japan), images transferred to computer using a high-resolution camera (BX51, Japan).

Morphmeterical methods were used to count DNs per unit area in CA1, CA3 and DG subdivisions of the hippocampus. The number of DNs was counted using a 10000 μm2 counting frame. The mean number of DNs per unit area (NA) in different regions of hippocampus was calculated using the following formula (33):

NA=∑Q̅a f . ∑P

In this formula “∑Q“ is the sum of counted particles appeared in sections, “a/f” is the area associated with each frame, and “∑P” is the sum of frame associated points hitting space.

*Statistical analysis*

The acquired data from BLL measurements and the DNs counting methods were reported as mean ± SE. For comparison of the lead blood level data obtained from before and after interventions in each group, paired sample t-test was used. The data resulted from pretreatment in each group of mothers was compared among all groups. The mean of lead blood level obtained at the end of experiments (after interventions) in mothers as well as their offspring at P50 in each group was compared in all groups. In addition, the mean number of DNs per unit area in each region of rat offspring hippocampus was compared in all groups. 

To compare the lead blood level and the number of DNs per unit area among all groups, at first, data normality was assessed by the Kolmogorov–Smirnov test and then one-way analysis of variance (ANOVA) was used followed by Tukey post hoc test using SPSS software version 11.5. P≤0.05 was considered as significant.

## Results

*Effects of garlic and ascorbic acid on blood lead levels*

**Figure 1:**
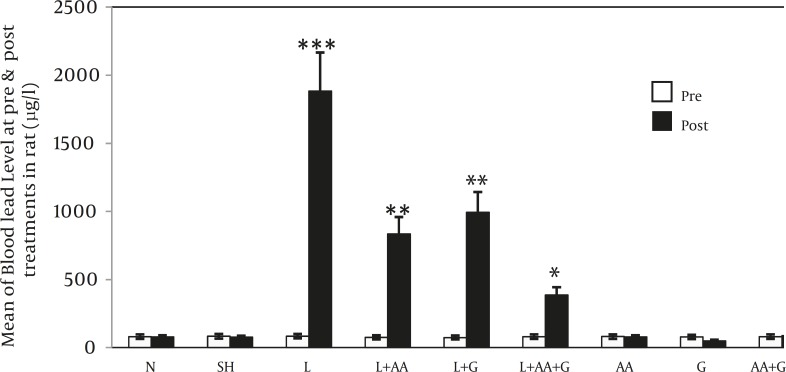
Comparisons of BLL in rats at pre and post treatment in each group (Mean±SE). Post-treatment BLLs in L, L+AA, L+G and L +AA+G groups increased significantly compared to each of their pretreatment (*** P<0.001, **P<0.01 and * *P*<0.05

**Figure 2:**
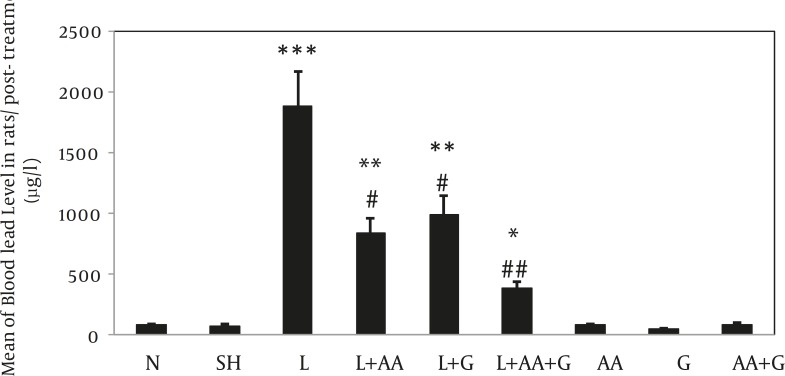
Evaluation of ascorbic acid and garlic effects on BLLs of rat at post- treatments in different groups (Mean±SE). BLLs increased in L, L+AA, L+G and L+AA+G groups compared to N and SH groups significantly (*** P<0.001, **P<0.05 and * P<0.01) and decreased in L+AA, L+G and L +AA+G groups compared to L group significantly (# *P*<0.05, ## *P*< 0.01) N= Normal, SH=Sham, L=lead, L+AA= Lead + Ascorbic Acid, L+G= Lead + Garlic, AA=Ascorbic Acid, G=Garlic, AA+G= Ascorbic Acid + Garlic

**Figure 3:**
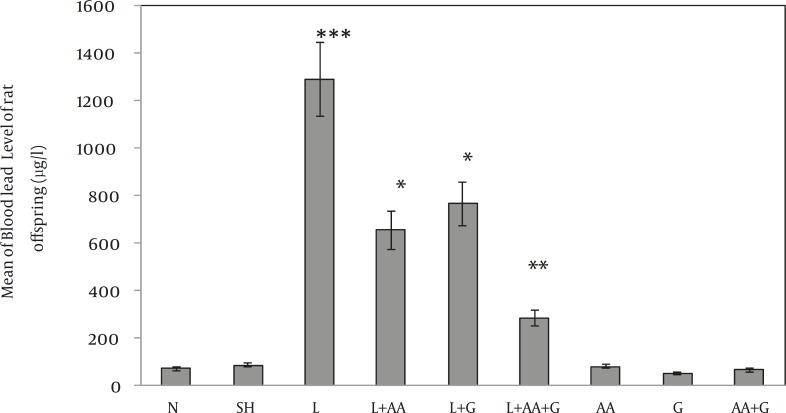
Evaluation of ascorbic acid and garlic effects on BLLs of rat offspring in different groups (Mean±SE). BLLs increased in L group compared to N, SH, AA, G and AA+ G groups significantly (*** P<0.001) and decreased in L+AA, L+G and L +AA+G Groups compared to L Group significantly (*P<0.05 and ** *P*<0.01). (N= Normal, SH=Sham, L = Lead, L+AA = Lead + Ascorbic Acid, L+G= Lead + Garlic, AA=Ascorbic Acid, G=Garlic, AA+G= Ascorbic Acid + Garlic

BLL measurements and data analysis in all animals groups were summarized as follows: 

a) There was no significant difference among all groups in comparisons of pretreatment blood lead levels.

 b) Comparisons of BLLs between pre and post treatment in each group showed significant increase at post-treatment in four groups including L, L+G, L+AA and L+AA+G (Figure 1) ( *** *P<*0.001,** *P <*0.01, * *P <*0.05) . 

c) Comparisons of post-treatment BLLs among all groups showed significant increase in four groups including L, L+G, L+AA and L+AA+G compared to N and SH groups (***P <0.001, ** *P<*0.01, * *P<*0.05 ). Nevertheless, BLLs in the L+G, L+AA, and L+G+AA groups were reduced significantly compared to the L group (#*P<*0.05, ## *P<*0.01) (Figure 2). 

d) Comparisons of rat offspring BLLs in different groups showed an increase in L group compared to N, SH, AA, G and AA+ G groups significantly (***P<0.001) and decreased in L+AA, L+G and L +AA+G groups compared to L group significantly (*P<0.05 and **P <0.01) (Figure 3).

*Effects of ascorbic acid and garlic on the number of lead-induced DNs per unit area in the rat offspring hippocampus*

**Figure 4:**
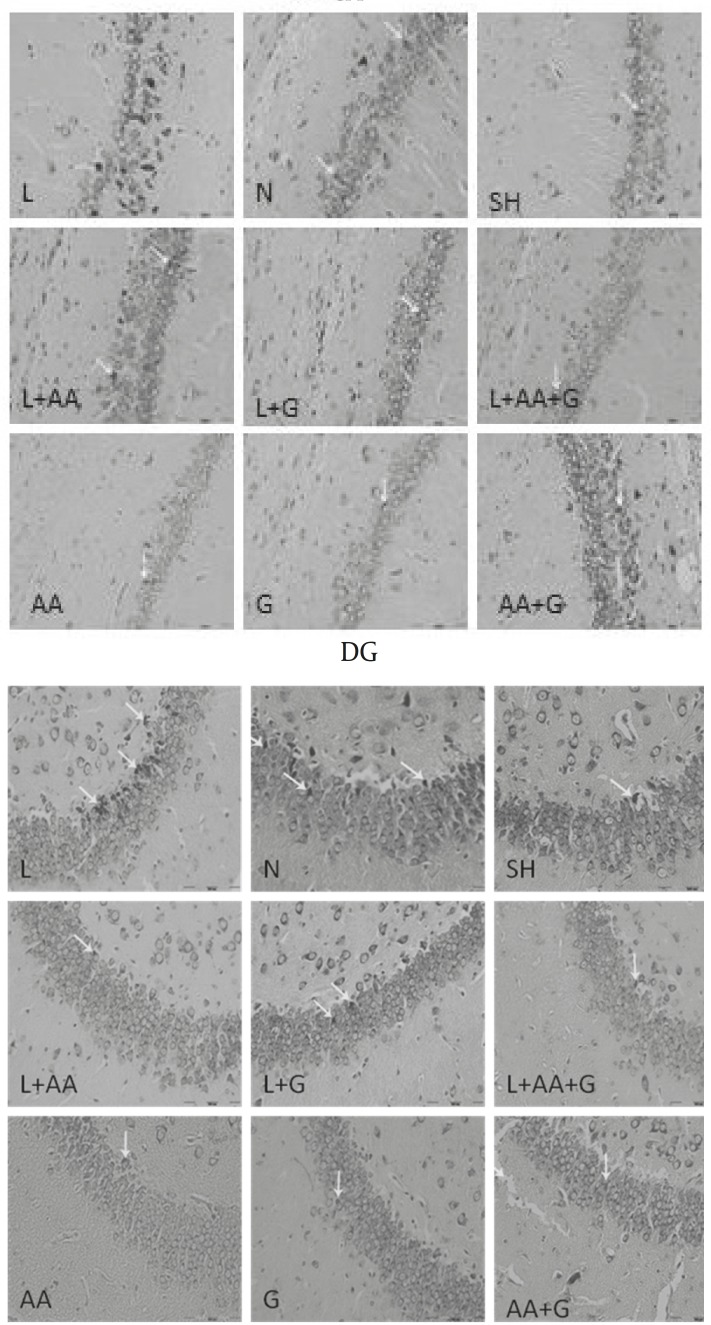
Photomicrographs show the DNs in CA1, CA3 and DG hippocampal subdivisions of rat offspring at P50, Toloidin Blue Stained in the Lead-Exposed (L), Lead + Garlic (L+G), Lead + Ascorbic Acid (L+AA), Lead + Ascorbic Acid + Garlic(L+AA+G), Ascorbic Acid(AA), Garlic(G), Ascorbic Acid + Garlic (AA+G), Normal (N) and Sham (SH) groups, Arrow= Dark Neuron, (×200)

**Figure 5:**
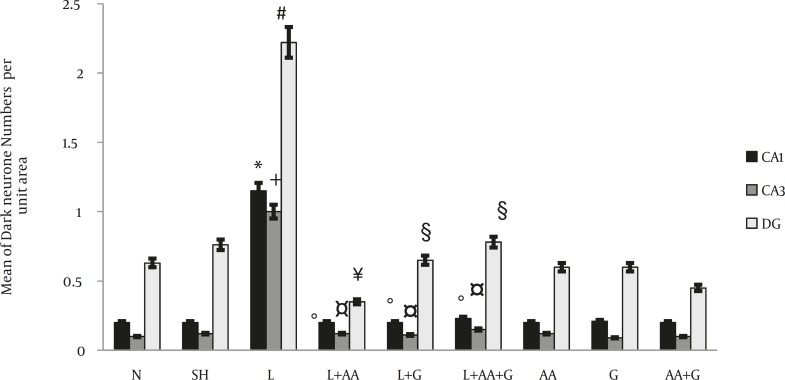
Evaluation of ascorbic acid and garlic effects on the number of lead- induced DNs per unit area in CA1, CA3 and DG regions. The DNs in CA1, CA3 and DG hippocampus subdivisions increased in the L Group significantly compared to control group (*P<0.01, +*P*<0.05, #*P*<0.05 respectively). The numbers of DNs in the L+G, L+AA and L+AA+ G groups were reduced significantly in the CA1, CA3 and DG regions of the hippocampus compared to the L Group (º*P*<0.01, ¤*P*< 0.01, ¥*P*<0.001, § *P*<0.01) N= Normal, SH=Sham, L=Lead, L+AA= Lead + Ascorbic Acid, L+G= Lead + Garlic, AA=Ascorbic Acid, G=Garlic, AA+G= Ascorbic Acid +Garlic

 The numbers of DNs per unit area (NA) of the CA1, CA3 and DG subdivisions of the hippocampus were counted. A few DNs were found in different regions of hippocampus in control group animals. In comparison to controls, the mean number of DNs per unit area in all subdivisions of hippocampus in clouding CA1, CA3 and DG was increased in L group significantly (*P<*0.01, *P<*0.05 and *P<*0.01, respectively). Nevertheless, the mean number of DNs in the L+G, L+AA and L+AA+ G groups was reduced significantly in the CA1, CA3 and DG regions of the hippocampus compared to the L group (Figure 4, Figure 5). The mean number of DNs in G, AA and AA+G groups were reduced in the CA1, CA3 and DG regions of the hippocampus compared to the control group but these differences were not significant.

## Discussion

The present study was undertaken to investigate the preventive effects of ascorbic acid and fresh garlic juice on lead- induced neuronal damage during rat hippocampus development. 

Lead can enter the CNS in circulation and induces negative effects, and there are some associations between the BLL and level in the brain tissue. Lead can disrupt the main structural components of blood brain barrier by damaging endothelial, glial cell and affecting the formation of tight junctions between barrier cells (11).

Our data showed that the BLL was increased significantly in rats receiving lead alone. BLLs revealed a significant and constant decreasing trend in the rats receiving garlic and lead, almost to the level recorded in normal rats.

Garlic juice contains sulfur-containing compounds like *S*-allyl cystine, S-allyl mercaptocystein and alliin which may have a chemoprophlylactic role to use in lead toxicosis. These compounds might act as lead chelator, enhancing its excretion in urine and also preventing gastrointestinal lead absorption resulting in reduced BLL (27-29).

In this work, the numbers of DNs per unit area (NA) were counted by means of morphological methods and analysed in different hippocampal subdivisions separately, as a marker of neurotoxic effects of lead. 

Our results demonstrated a significant increase in the number of DNs in all hippocampus subdivisions of lead exposed group when compared to controls. This study results clearly revealed that lead exposure during development was able to produce DNs in CA1, CA3 and DG regions of young pup’s hippocampus, as a marker of neurotoxic effects of lead, especially in growing states.

In former studies, researchers described three major categories for toxicity of lead. The primary toxicity of lead derives from its ability to cause oxidative stress by inducing the generation of ROS, reducing the antioxidant defense system of cells via depletion of glutathione, inhibiting sulfhydryl-dependent enzymes and/or increasing susceptibility of cells to oxidative attack (33-36). The second group of toxicity effects results from its chemical similarity to calcium. This similarity allows lead access to critical cellular pathways, particularly within the mitochondria and in second messenger systems, where it competitively antagonizes calcium action (37). This action of lead affects calcium-dependent processes which include metal transport, energy metabolism, apoptosis, ionic conduction, cell adhesion, inter- and intracellular signaling and protein maturation (38, 39). Thirdly, lead appears to affect nucleic acids by an unknown mechanism, raising concern about chromosomal abnormalities and genetic regulation (40, 41). 

We also found a markedly increase in the number of DNs in all hippocampal subdivisions of lead exposed group in comparison to ascorbic acid and fresh garlic juice treated groups as well as sham and control groups.

Although, the exact mechanisms of the effect of ascorbic acid are not completely known, it has a role in attenuation of oxidative cell death, inhibition of FAS-induced apoptosis and modulation of genomic protection through the quenching of intracellular reactive oxygen species (ROS).

Today’s, chelating agents are the best therapeutic strategy for the management of lead toxicity (42). The most commonly chelating agents are calcium disodium ethylene diamine tetraacetic acid (CaNa2EDTA), succimer (2, 3-meso-dimercaptosuccinic acid) and d-penicillamine. While these agents reduce BLLs and increase the urinary excretion of the metals, their safety and efficacy are less established. In addition, these agents are generally nonspecific regarding their affinity for metals. There are evidences that some nutrients, especially ascorbic acid, exhibit some protective effects against lead intoxication (42, 43). 

Garlic extract is an antioxidant candidate because of scavenging reactive oxygen species, enhancing cellular antioxidant enzymes superoxide dismutase. Besides these properties, the other efficiency of garlic is perhaps due to the presence of these sulfur-containing amino acids and compounds having free carboxyl (C=0) and amino (NH2) groups in their structures. These biologically active compounds might have chelated lead and enhanced its excretion from the body resulting in reduced lead accumulation in soft tissues and blood (21).

In conclusion, this study results indicated that the lead poisoning, especially during pregnancy and lactation, the critical period of brain development, can induce the production of DNs in hippocampus of young rats. These cellular alterations could be a reason for the behavioral and cognitive impairments observed in developmentally lead exposed offspring. Moreover, the fresh garlic juice as well as ascorbic acid showed preventive and beneficial effects in lead induced production of DNs in hippocampus.

## Conclusion

In conclusion, this study results indicated that the lead poisoning especially during pregnancy and lactation, the critical period of brain development, can induce the production of DNs in hippocampus of young rats. These cellular alterations could be a reason for the behavioral and cognitive impairments observed in developmentally lead exposed offspring. Moreover, the fresh garlic juice as well as ascorbic acid showed preventive and beneficial effects in lead induced production of DNs in hippocampus.
